# Cooperation in microbial communities and their biotechnological applications

**DOI:** 10.1111/1462-2920.13767

**Published:** 2017-05-29

**Authors:** Matteo Cavaliere, Song Feng, Orkun S. Soyer, José I. Jiménez

**Affiliations:** ^1^ School of Informatics, BBSRC/EPSRC/MRC Synthetic Biology Research Centre University of Edinburgh Edinburgh EH8 9AB UK; ^2^ Center for Nonlinear Studies Theoretical Division (T‐6), Los Alamos National Laboratory Los Alamos NM 87545 USA; ^3^ School of Life Sciences, BBSRC/EPSRC Warwick Integrative Synthetic Biology Centre University of Warwick Coventry CV4 7AL UK; ^4^ Faculty of Health and Medical Sciences University of Surrey Guildford GU2 7XH UK

## Abstract

Microbial communities are increasingly utilized in biotechnology. Efficiency and productivity in many of these applications depends on the presence of cooperative interactions between members of the community. Two key processes underlying these interactions are the production of public goods and metabolic cross‐feeding, which can be understood in the general framework of ecological and evolutionary (eco‐evo) dynamics. In this review, we illustrate the relevance of cooperative interactions in microbial biotechnological processes, discuss their mechanistic origins and analyse their evolutionary resilience. Cooperative behaviours can be damaged by the emergence of ‘cheating’ cells that benefit from the cooperative interactions but do not contribute to them. Despite this, cooperative interactions can be stabilized by spatial segregation, by the presence of feedbacks between the evolutionary dynamics and the ecology of the community, by the role of regulatory systems coupled to the environmental conditions and by the action of horizontal gene transfer. Cooperative interactions enrich microbial communities with a higher degree of robustness against environmental stress and can facilitate the evolution of more complex traits. Therefore, the evolutionary resilience of microbial communities and their ability to constraint detrimental mutants should be considered to design robust biotechnological applications.

## Evolutionary dynamics and cooperation in microbial populations

The design and optimization of microorganisms for biotechnological purposes often considers cells in isolation. While this reductionist approach aims to thrive for simplicity in the process, it creates a situation that rarely takes place in Nature. In their natural environment microorganisms thrive in complex communities in which the fitness of a single cell depends on the interactions with other cells in the population (West *et al*., 2006). This scenario also applies to bioprocesses in which the efficiency of the process is coupled to the production of shared (public) goods that allow cells to perform tasks in a ‘cooperative’ manner (Lindemann *et al*., 2016): a good example of shared goods are the cellulases secreted in the production of cellulosic ethanol (Zomorrodi and Segrè, 2016).

The presence of cooperative interactions has a significant impact on the evolutionary dynamics of microbial communities, represented by the change in the frequencies of cells and species that implement different physiological strategies (such as production of public goods vs. not). Thus, cooperative traits need to be taken into account when using an evolutionary approach for optimizing a given bioprocess. It is possible that simple selection schemes targeting a bioprocess‐related trait (e.g. growth rate) will not align with the selection for the cooperative trait (e.g. production of costly extracellular enzymes) ultimately resulting in the loss of the trait. Indeed, tradeoffs between the optimization of so‐called high‐rate and high‐yield strategies are frequently observed in controlled evolutionary experiments (Bachmann *et al*., 2013). Thus, we advocate considering the interactions between the cells and the functioning of cooperative traits when designing evolutionary optimization and stabilization of bioprocesses. Achieving this would require considering how ‘social’ interactions shape microbial processes, rather than simply focusing solely on individualistic traits such as growth rate.

This situation may confront the intuitive idea that ‘evolution implies improvement’ (i.e. the average fitness of the community is expected to increase over generations as it would be expected for monocultures). The key point is that the presence of interactions between the species gives rise to a more complicated evolutionary picture in which the fitness of a cell depends not only on its phenotype but also on the overall composition of the population. The spreading of a given phenotypic trait may thus change the fitness of other members of the community and these changes may in turn feedback on the fitness of the individual cells (West *et al*., 2006). These intertwined selection mechanisms are expected to operate in any microbial population where there is possibility of different cells implementing different strategies with respect to their physiology, as is the case of phenotypic heterogeneity.

Phenotypic heterogeneity arises even in monocultures and simple bioprocesses due to different reasons, such as the use of non‐homogenous culture conditions, stochasticity in gene expression and differential epigenetic control (Enfors *et al*., 2001; Avery, 2006; Müller *et al*., 2010). Such heterogeneity does not represent a static picture – cells communicate, compete and cooperate and the success of a trait may be consequence of the interaction with the other traits and of the specific ecological context (Carlquist *et al*., 2012). Therefore, it is not sufficient for a trait to be successful in one specific setting but rather, it needs to be successful given the presence of other traits and the associated ecological context. Moreover, the dilution of a trait may lead to changes in the community (both ecological and/or in the frequency of other traits) that could feedback on the evolutionary dynamics of the trait itself. For instance, a trait may be favoured by natural selection only when rare in a complex population, becoming disfavoured when it is more frequent. These complex evolutionary and ecological dynamics, in which the success of a trait depends on the composition of the community, can be mathematically analyzed with evolutionary game theory (Nowak and Sigmund, 2004; Frey, 2010).

Evolutionary game theory is a mathematical framework that comes from classical game theory used to describe the behaviour of rational players. Classical game theory tries to analyse the behaviour in conflicts in economic and social settings in which the success of an individual strategy depends on the strategies employed by the other players. A well‐studied example in game theory is the prisoner's dilemma in which the choices to either confess or remain silent determine whether two suspects are considered guilty (Axelrod, 1990). In evolutionary game theory, the strategies are not associated to rational and cognitive choices, but are traits encoded into inherited programs that can be passed to the offspring (for this reason, the terms trait and strategies are used in an indistinguishable manner). Traits such as the usage of metabolic pathways or the expression of certain enzymes can be then regarded as strategies and a successful strategy is then selected for.

In a microbial community composed of species that compete using different strategies, each of the individual cells possesses a fitness that depends on its strategy and on the strategy of the individuals with whom it interacts. Individuals that use more successful strategies have higher chances to propagate and their frequency in the community will increase. Although the dynamics of an evolutionary game theory model can be studied analytically when the set of strategies is small, due to the large number of interactions taking place in microbial communities many authors prefer to simulate the dynamics of the community using agent‐based modelling. In these models, the replication and death of individual cells (agents) are explicitly simulated using a system updated by a series of discrete events (Adami *et al*., 2016). These types of models also include the possibility of adding mutations that can introduce novel strategies not yet present in the species, which can be used to simulate random evolution of members of the community (Eriksson and Lindgren, 2005).

In cellular populations, a cooperative trait is often characterized by the presence of a shared public good, which is a finite resource, produced by cooperative cells and that is freely available to all other cells. The presence of a public good is always associated with the risk of cheating cells, which exploit the public good without providing any contribution to it and which can spread in the population – due to their improved fitness arising from not investing the costs associated with public good production. Although in this review we focus on microbial populations, this is a very general issue in the sustainability of many organisms at different scales including humans, justifying why the evolution (and resilience) of cooperation is considered one of the major open questions in biology (Pennisi, 2009).

**Figure 1:**
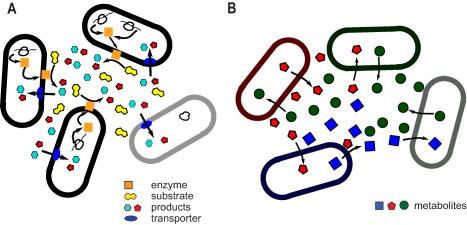
A. Interactions based on shared public goods. Some cells (cooperators, shown in black edge) produce an enzyme required to split a substrate into digestible products. Other cells (cheats, shown in grey), do not produce the enzyme but take advantage of the public goods produced by the others. B. Interactions based on cross‐feeding. Some cells in the community excrete metabolites that can be taken up by other cells giving rise to a web of interactions.

Evolutionary conflicts between cooperative and cheating cells have been studied in a variety of microbial scenarios, including the conversion of sucrose into glucose by the yeast *Saccharomyces cerevisiae* (Gore *et al*., 2009), the production of the shareable iron‐scavenging siderophore pyoverdine in *Pseudomonas aeruginosa* (Kümmerli *et al*., 2009) and the formation of fruiting bodies in *Myxobacteria* (Velicer and Vos, 2009). Given the potential similarities with cellulose and other polymers biodegradation, the example from yeast is worth explaining further. In this case, cooperative and cheating cells only differ by the production of the enzyme invertase that converts sucrose into glucose and fructose. Both monosaccharides can eventually diffuse away from the producing cell and become available to neighbouring cells. In other words, they become public goods: cooperators – the cells that ‘feed’ themselves and their neighbours at the expense of expressing the enzyme – can be exploited by cheaters, cells that do not express the enzyme and rely on cooperators to make food (Fig. 1A). In a scenario like this, it would be expected that cheaters could take over the population. However, the fitness of the cells is a nonlinear function of the glucose concentration and, for certain values of glucose uptake and metabolic cost of enzyme production, it is possible to observe the coexistence of the two species as anticipated by an evolutionary game theory model (Gore *et al*., 2009). In fact, in a complex community composed of multitude of species it is likely that such mechanistic properties relating to the implementation of the different strategies, such as regulatory mechanisms controlling the production of a public good, will affect the evolutionary and ecological dynamics of the strategies and thus the whole community. Before discussing further these potential mechanisms that can stabilize cooperative interactions, we will first describe types of cooperative interactions in microbial communities.

## Microbial cooperations based on public goods

Shared (public) goods are molecules produced by certain individuals and can benefit the entire population (West *et al*., 2007). As explained above, these molecules are synthesized at a cost and, therefore, are susceptible to be exploited by cheater cells that can benefit from them but do not contribute to their production – hence acquiring a fitness advantage over cooperators. This type of cooperation is based on a large variety of shared molecules: siderophores, enzymes, biosurfactants, components of biofilm matrix, quorum sensing molecules, bacteriocins (proteins secreted by one strain to inhibit the growth of a closely related strain) and toxins as summarized in (West *et al*., 2007). Given their interest in microbial biotechnology, in this review we will focus on the secretion of degradatory enzymes.

Microorganisms digest large macromolecules, which are poorly soluble, through the secretion of extracellular enzymes. The macromolecules are typically polymers of biological or synthetic origin, such as starch, cellulose and polyesters, which constitute an abundant source of nutrients for bacteria, fungi and other eukaryotic microorganisms (Allison, 2005; Richards and Talbot, 2013). These polymers also constitute a very interesting substrate for industrial bioprocesses, as they are inexpensive, biodegradable at some extent and often obtained from renewable sources (Gross and Kalra, 2002). The enzymes secreted by microorganisms act by degrading the macromolecules into simpler and smaller components that can then be assimilated by the microbial community (Burns, 2010). In this scenario, the dynamics of the cooperating and cheating populations depend on parameters such as the cost of producing the enzymes and their diffusibility (Allison, 2005).

Cellulases and oxidative enzymes secreted to cleave cellulose such as cellobiase dehydrogenases can be considered as instances of ‘public goods’ (Dimarogona *et al*., 2012) and are found in the genome of most wood‐degrading microbial communities (Zamocky *et al*., 2006). Similar to cellulases, amylases capable of degrading the glycosidic linkages of starches also play an important role as public goods and have been identified in many bacteria and fungi, such as *Bacillus subtilis* (Coleman and Elliott, 1962), *Thermomyces lanuginosus* (Arnesen *et al*., 1998), *Penicillium expansum* (Doyle *et al*., 1998) and several species of *Streptomyces* (El‐Fallal *et al*., 2012). Similarly, enzymes responsible for the digestion of other macromolecules such as extracellular lipases and proteases are also examples of public goods, and their production in a complex microbial community is influenced by the interactions between its members (Willsey and Wargo, 2015). Collectively produced enzymes are also responsible for the degradation of oil‐derived plastic polymers such as poly‐ethyleneterephthalate (PET). The identification of bacterial species producing enzymes capable of PET depolymerization, therefore generating molecules that can then be assimilated by the microbial community in that niche (Chen *et al*., 2010; Yoshida *et al*., 2016) paves the way for the remediation of PET waste and its use as a bioprocessing substrate (Wierckx *et al*., 2015).

## Microbial cooperations based on metabolic interactions

Metabolic exchange is another way in which microorganisms can interact cooperatively. Metabolic interactions are widespread in natural microbial communities and arise from metabolites from one species being used as energy sources or building blocks by other species (Paczia *et al*., 2012; Cooper and Smith, 2015; Fiore *et al*., 2015). The former scenario leads to cross‐feeding, whereas the latter can lead to emergence of auxotrophies (an organism fully relying on the environmental provision of certain compounds required for its growth) (Fig. 1B). The metabolites released into the environment can be explained by either passive or active means, i.e. organisms not being able to maintain certain compounds due to leakage issues or actively secreting those compounds due to some functional benefits. While the former explanation could arise due to some fundamental biophysical limitations on biological membranes, the second (functional) explanation is difficult to justify within a simplistic view of organismal fitness. One could naively argue that since other organisms use the secreted metabolites as a resource, evolution should have allowed the ‘secreting organism’ also to innovate the capacity of using this metabolite (as an energy source or building block) rather than secreting it. This naïve view, however, ignores limitations arising from cellular tradeoffs and thermodynamics.

### Metabolic interactions emerging from thermodynamic limitations

**Figure 2:**
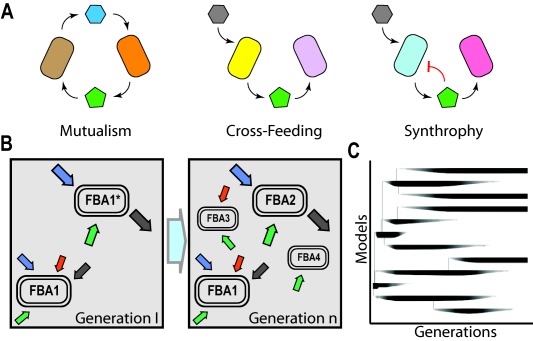
A. Metabolic interactions that can take place in a population. Cells can exchange metabolites that are required to support each other's growth in a mutualistic interaction (left). One of the cells can use a metabolite excreted by another cell, favouring in this way the metabolism of the producer through the pathways leading to the excretion (centre). When the metabolites excreted have an inhibitory effect on the producer (e.g. because they lead to thermodynamic equilibrium), the relationship with a degrader cell of the inhibitory metabolite is mutually beneficial and known as syntrophy (right). B. Dynamic modelling of the evolution of Flux Balance Analysis models. Cells can be modelled as metabolic networks exchanging metabolites with other cells in the population. In this abstraction each cell is represented by a FBA model. These models can replicate over time and also evolve, producing populations composed by models with different constrains for uptake and secretion of metabolites. C. Dynamic analysis of model genealogy. The frequency of each model in the population changes over time being the darkest bars the most abundant models. Due to mutations, new models arise and they are represented as new branches in the phylogeny. Plot redrawn from Großkopf *et al*. (2016).

In principle, cross‐feeding and auxotrophic interactions could be seen as an extreme form of cooperation (i.e. ‘altruism’) as they benefit only the receiving organisms. Under certain conditions, however, secretion of internal metabolites can also benefit the producer leading to a mutually‐beneficial interaction: if the products released have an inhibitory effect on the producer, the presence of an additional species that would assimilate these products would lead to more mild forms of cooperative interaction rather than a straight ‘altruistic’ act on behalf of the producer (Lilja and Johnson, 2016). More specifically, this type cross‐feeding interaction, involving release of inhibition arising from byproducts of metabolism of one organism by another is often referred to as syntrophy (Fig. 2A). The most‐well known example is the H_2_‐mediated syntrophic interactions between secondary degraders and methanogens (Schink, 1997). In these interactions, the inhibition of the degrading species arises due to its growth‐supporting metabolic reaction reaching towards thermodynamic equilibrium as H_2_ accumulates (Schink, 1997; Großkopf and Soyer, 2016). This ‘thermodynamic inhibition’ is relieved by the consuming of H_2_ by the syntrophic partners (McInerney and Bryant, 1981; Seitz *et al*., 1988; Scholten and Conrad, 2000), creating a situation in which continued growth is only possible when the two partners coexist. Many of the biodegradation processes consist of individual syntrophic and cross‐feeding interactions among different species (Schink, 1997), with examples including the degradation of monoaromatic and polyaromatic compounds in syntrophy with methanogens (Knoll and Winter, 1989; Berdugo‐Clavijo *et al*., 2012; Morris *et al*., 2013). Syntrophic interactions are also important in oil‐degrading microbial communities, although the exact roles of many individual members in these communities are less clear. It has been reported, for instance, that syntrophic interactions between *Desulfatibacillum alkenivorans* and *Methanospirillum hungatei* are necessary to degrade refractory hydrocarbons (Westerholm *et al*., 2011; Callaghan *et al*., 2012).

These examples illustrate how ubiquitous and essential syntrophic interactions are for complete degradation of organic compounds. Therefore, for fully being able to optimize bioprocesses and biotechnologies around organic degradation and transformations we need a better understanding of the emergence and maintenance of metabolic cooperations. It is important to note that syntrophic and cross‐feeding interactions are shown to alter cellular metabolic fluxes within individual species, as well as in simple communities such that the presence of a downstream syntrophic partner can result in changes in the metabolic by‐products and yields from upstream producer microorganisms (McInerney and Bryant, 1981; Seitz *et al*., 1988; Schink, 1997; Scholten and Conrad, 2000). In other words, organisms’ preferred metabolic routes (or ‘strategies’) would change with local substrate/product availabilities (as well as internal constraints such as on uptake rates or cofactor availabilities), but these in turn would depend on what other organisms would choose to do metabolically. From a theoretical perspective, this situation cannot be analyzed assuming a simple individual fitness optimization under constant selection pressure, but would require instead the combination of evolutionary game theory and ecology to develop theoretical frameworks and experimental model systems accounting for the described complex interplays.

The inclusion of thermodynamics in models of microbial growth and metabolism could contribute to unravel the emergence of metabolic interactions. Taking into account the thermodynamic constraints of growth‐supporting microbial biochemical reactions would enable better capturing changes in the concentrations of different compounds in the environment and thus allow direct linkage between ecology and individual growth rates. There have been several recent attempts in this direction, and models including the thermodynamics of metabolic reactions have been successfully employed to describe the dynamics of some biodegradation processes, such as the fermentation of glucose and the reduction of nitrate (González‐Cabaleiro *et al*., 2013, 2015; Cueto‐Rojas *et al*., 2015), to explain microbial diversity (Großkopf and Soyer, 2016), as well as to model individual species growth (Hoh and Cord‐Ruwisch, 1996; Jin and Bethke, 2007). Additional works in this direction will allow better predictive models to explain evolutionary and ecological dynamics of microbial communities under conditions where thermodynamics‐driven metabolic interactions dominate.

### Metabolic interactions emerging from cellular tradeoffs

As discussed above, fitness optimization is a complex function of multiple traits and it is subject to intrinsic tradeoffs that could readily explain metabolic secretions. In particular, the optimization of ATP‐generating pathways under limitations on enzyme investment and internal metabolic concentrations is shown to lead to the evolution of impartial pathways and metabolite excretion (Pfeiffer and Bonhoeffer, 2004). Similarly, limitations on membrane space and internal resources such as enzymes and conserved moieties can cause tradeoffs in substrate uptake rates and internal metabolic fluxes, resulting in different genotypes that differentially utilize respiratory (i.e. pathways ending with inorganic terminal electron acceptors) and fermentation (i.e. pathways ending with organic terminal electron acceptors) pathways (Majewski and Domach, 1990; Vemuri *et al*., 2006; Molenaar *et al*., 2009; Zhuang *et al*., 2011; van Hoek and Merks, 2012; Flamholz *et al*., 2013; Basan *et al*., 2015). Since the end products of fermentative pathways are usually still able to sustain further microbial growth, this could again explain the first stage of formation of metabolic interactions through metabolic excretions. Subsequently, limitations on substrate uptake are predicted to act as a force to drive metabolic specialization on such excreted compounds (Doebeli, 2002; Spencer *et al*., 2007).

The idea of cellular tradeoffs driving the emergence of metabolic cross‐feeding has recently been evaluated in a combined *in silico* and experimental evolution study (Großkopf *et al*., 2016). In that study, the authors have incorporated tradeoffs in a stoichiometric metabolic model of *Escherichia coli* by imposing global constraints on the total uptake rates. This model was then simulated using dynamical flux balance analysis, which allows modelling of both microbial growth and environmental substrate concentrations, and mutations, which can alter the distribution of total uptake flux among different substrates. In other words, this approach combined simulation of ecological and evolutionary dynamics at the same time; starting from a single model, the *in silico* simulations can lead to alterations both in the environmental conditions and mutant models (Fig. 2B). The application of this approach to the modelling of the experimental long‐term evolution of *E. coli* revealed that the combination of tradeoffs and ecological/evolutionary dynamics results in the emergence of two dominant models (Fig. 2C). These two models have distinct uptake fluxes suggestive of a cross‐feeding interaction; one model had increased glucose uptake and acetate excretion rate and the other had increased acetate uptake rate (Großkopf *et al*., 2016). Further experimental analyses revealed that the two models show metabolic flux patterns that qualitatively match experimentally observed genotypes in one lineage of the long‐term experiments, indicating that this approach might provide useful insights into how ecological and evolutionary dynamics can shape metabolic systems. Indeed, an emerging trend in the analysis of community dynamics is to increasingly combine multi‐species ecological simulations with stoichiometric models describing the metabolism of those interacting species in an attempt to generate insights into ecology – evolutionary interplays (Louca and Doebeli, 2015; Widder *et al*., 2016; Zomorrodi and Segrè, 2016).

## Factors contributing to the stabilization of cooperative interactions in microbial populations

### Structured environments

**Figure 3:**
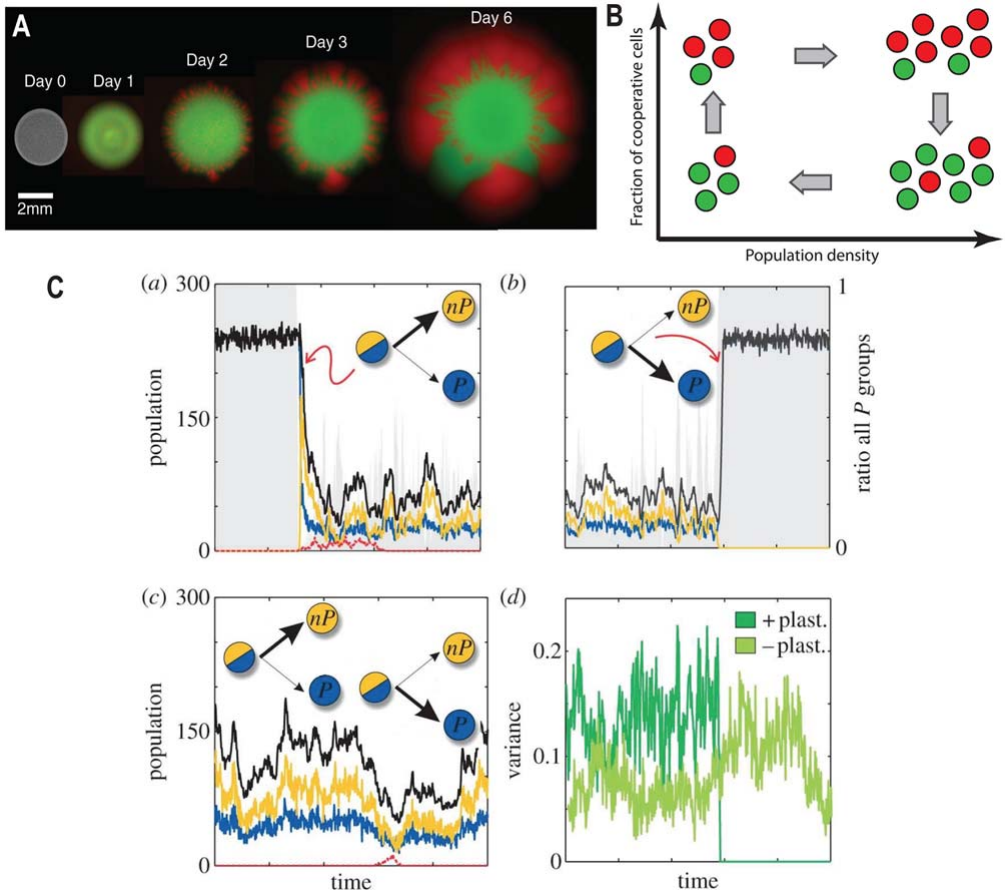
Mechanisms to Preserve Cooperation in Cellular Communities. A. A structured environment can facilitate cooperation. The figure shows the growth of fluorescently labelled colonies (cooperators in red, cheaters in green) of *S. cerevisiae* (Figure from (Van Dyken *et al*., 2013)). Cooperative cells produce invertase that breaks down sucrose into digestible glucose and fructose. Non‐producers cells (cheaters) have a fitness advantage because they do not produce invertase but can access glucose. In unstructured environment (liquid culture) cooperators decline. However, in a spatial environment (obtained by spotting a droplet of mixed cooperator/cheater cultures onto solid medium) cooperators can spread over cheaters. The diffusion of cells leads to the formation of discrete sectors – cooperator sectors are more productive than cheater sectors and expand radially faster. B. Eco‐evo dynamics can preserve cooperation in communities of *S. cerevisiae* (redrawn from (Sanchez and Gore, 2013)). Red circles represent cooperative cells (invertase producers), green circles represent cheaters (non‐producers). Below a certain cooperator density, there is little glucose available. Cooperative cells grow at a slow rate on the little amount of glucose they can retain, while cheater cells grow more slowly (it is crucial that cooperators have preferential access to the glucose). Above a certain cooperator density, both cooperators and cheaters grow at a fast rate because of the large pool of available glucose, but cheaters grow faster as they do not have the burden of producing invertase. Such density‐dependent selection favours cooperators at low densities and cheaters at high densities, which leads to the stable coexistence of cooperative and cheating yeast cells. C. Regulation of public good production can preserve cooperation in a meta‐population model in which the population is transiently divided in sub‐populations (figure from (Cavaliere and Poyatos, 2013)). *In‐silico* simulations present two possible successful types of regulation against cheaters: positive plasticity (top row) in which cooperators constraint cheaters by stopping the production of public good when cheaters appear (a) and fully restarting only when cheaters have disappeared (b) and negative plasticity (bottom row) in which cooperators produce permanently low amounts of public good which helps controlling cheaters invasion (c). Thick arrows denote the cellular decision to produce (P) or not produce (nP) the public good. The success of the regulation is coupled to the heterogeneity (variance) of the subpopulations, i.e., positive plasticity transiently modifies the variance while negative plasticity keeps a relatively constant heterogeneity (variance shown in (d) correspond to trajectories (b) and (c) respectively).

One of the basic mechanisms that affect the resilience of cooperation is the presence of spatial structure. Structure would ultimately facilitate the resilience of cooperation as it allows the ‘segregation’ of cooperative from cheating cells (Nowak, 2006) (i.e. cooperative cells can then share the produced public good with the similar trait, excluding cheating cells) (Fig. 3A).

There are several theoretical studies and experimental evidences of spatial segregation in cellular populations (Van Dyken *et al*., 2013), with biofilms being a paradigmatic example of bacterial communities exhibiting stable cooperation due to the segregation in structured environments (Nadell *et al*., 2009). The structure and composition of biofilms can feedback on the highly dynamic competition between sub‐populations of cooperators (i.e. contributing to the biofilm assembly) and cheaters. In these circumstances, the spatial arrangements of the distinct genotypes crucially affect the degree of cooperation and competition present in the biofilm (Nadell *et al*., 2016).

A broader notion of structure can also refer to the case of having a population distributed into different heterogeneous sub‐populations that may be spatially segregated (e.g. forming colonies). In this case, the structure of the population can lead to a characteristic issue of multi‐level selection known as Simpson's paradox. Simpson's paradox is a statistical phenomenon that can emerge when comparing groups of data; groups can display a trend when analysing them individually, but this trend is reversed when the groups are combined. A famous example of Simpson's paradox is the one behind the gender discrimination accusation against the University of Berkeley in early 1970s. In that case, 44% of the total male applications to the graduate school were accepted against the 35% of the female applicants suggesting a bias against female applicants. Looking into how the applications were distributed among the different departments, however, it became clear that there was no bias, and the differences in the rates were the result of a majority of women having applied to the most competitive departments, which decreased the success rate of the female applicants. In other words, the apparent bias is only the result of the ways the applications are aggregated together (Bickel *et al*., 1975). In the context of microbial communities, Simpson's paradox is shown to emerge when the different sub‐groups are sufficiently heterogeneous in their composition to guarantee that in the aggregate population the cooperative individuals have an advantage over the cheating cells (despite in each of the colonies – the disaggregated population – cheaters are favoured) (Chuang *et al*., 2009). This finding suggests that the opportune design of the organization of a microbial community in sub‐populations (and subsequent coalescence of those sub‐populations) may be useful to improve its resilience to detrimental mutants. In general, other more complex notions of structured populations from ecology (e.g. meta‐population dynamics) could also be relevant to understand and control the evolutionary dynamics of cooperative interactions (Datta *et al*., 2013).

### Interplay between ecological and evolutionary dynamics

Another stabilizing and driving factor beyond cooperative interactions in microbial communities is the interplay between ecological and evolutionary dynamics that results in changes in the composition of the community over time. This happens when, due to the interactions in a community, certain traits (such as cheating and cooperation) are selected for or against, resulting in rapid changes in the frequency of the individuals carrying the trait that affect the ecology of the global community. The changes in the ecology can then feed‐back on the selective advantage of the different traits (as discussed above), leading to an eco‐evolutionary feedback (Fig. 3B) (Lennon and Denef, 2015). This aspect has become of recent interest due to several theoretical and experimental studies showing the non‐trivial effects of the time‐scales overlap between ecology and evolution in what are called eco‐evo feedbacks (Schoener, 2011). There are several examples of eco‐evo feedbacks in microbial populations investigated experimentally (Fiegna and Velicer, 2003; Ross‐Gillespie *et al*., 2009; Moreno‐Fenoll *et al*., 2017) with the best known example being the interplay between population density and fitness (Sanchez and Gore, 2013). For instance, in the yeast communities discussed above, cooperative cells have higher fitness than cheating cells only at lower population density. This, coupled to the fact that cheaters lead to lower population growth, facilitates the observed coexistence between the two traits, i.e. the stabilization of cooperation (Sanchez and Gore, 2013). Eco‐evo feedbacks can be modelled by adding notions of population dynamics to evolutionary game theory, leading to the framework of ecological public good games (Hauert *et al*., 2008) that extend the standard evolutionary game theory (in which, usually, the focus of the analysis is the change in frequency of a certain trait). Combination of population dynamics with metabolic models at the level of individual species or genotypes (Harcombe *et al*., 2014) with evolutionary dynamics (Großkopf *et al*., 2016) is another promising route towards capturing eco‐evolutionary dynamics, especially when cooperative interactions involve metabolite secretions.

### Regulatory mechanisms

Another potential factor for the stabilization of cooperation that has recently attracted attention is cellular regulatory mechanisms. Animals, including humans, have developed complex social strategies to control cheaters, and there is great interest in determining to which extent single cell organisms could employ similar mechanisms to fight detrimental mutants (Travisano and Velicer, 2004).

One of these regulatory mechanisms is known as ‘reciprocity’. In this case, the amount contributed of a public good depends on the environmental conditions, which in turn may depend on the contributions made by others. This is for instance the case of iron uptake in *P. aeruginosa* where iron scavenging siderophores (the public good) are released in greater or smaller quantities depending on the amount of iron in the environment (Kümmerli *et al*., 2009). Recent experiments using this system have confirmed that cells use a type of ‘reciprocity’ that facilitates the control of cheaters: the cellular decision of producing public good is made only in an environment with many producers. In other words, the cells seem to implement a rule stating ‘cooperate when surrounded by mostly cooperators’. Coupled to quorum sensing, this rule allows bacteria to match their investment at lower levels of population structuring and it is an effective way to repress cheaters (Allen *et al*., 2016). In yeast, a similar mechanism happens in the production of invertase. Another regulatory mechanism that could be interpreted as a functional ‘decision’ to limit the spread of cheaters is to increase the noise in the expression of genes encoding for public goods (Gore *et al*., 2009). This is the case of self‐destructive cooperation, in which cooperative cells die while helping others, for example, as it happens during the secretion of toxins that enhance the colonization of tissues by certain bacterial pathogens (Ackermann *et al*., 2008). Since the toxin is genetically encoded, it is only expressed by a fraction of the population or the whole microbial population would die. The ‘decision’ on which cells make the ultimate sacrifice is given by the stochastic expression of the gene encoding the toxin. Similarly, cell‐cell variability in the production of other kinds of public goods may allow cooperative cells to temporarily switch off the production of a public good, therefore limiting its cost and allowing for enhanced competition against the cheating cells.

These types of cellular decision‐making mechanisms can interplay with an underlying eco‐evo dynamics (Harrington and Sanchez, 2014) and crucially affect the resilience of cooperation, as shown in theoretical models (Cavaliere and Poyatos, 2013) (Fig. 3C). Thus, it is plausible to propose the control of public good production for successful bioprocesses (such as the described cellulose degradation) through existing gene regulatory mechanisms or by engineering such mechanisms *de novo*.

### Horizontal gene transfer of cooperative traits

Mobile genetic elements (plasmids, bacteriophages, transposons, etc.) transmitted via horizontal gene transfer are one of the main factors contributing to shaping microbial evolution. Apart from the genes essential for replication and transmission, mobile elements often carry multiple traits that enable social interactions in microbial communities and make them active agents defining the evolutionary dynamics of these communities (Rankin *et al*., 2011).

Cooperative traits such as public good producing exoenzymes are commonly acquired due to the transference of mobile elements. In fact, a genomic analysis in some bacterial species show that the frequency of genes encoding extracellular proteins is significantly higher in chromosomal locations known to be transferred (e.g. transposons) compared to regions that are not, and this frequency is even higher in plasmids, which were the most mobile elements present in the analysis (Nogueira *et al*., 2009). Horizontal gene transfer is also responsible for the transmission of exoenzymes in eukaryotic microorganisms, as revealed by a similar analysis carried out in osmotrophic fungi, in which it became evident that not only the enzymes, but also the transporters required for the uptake of the products resulting from the activity of the enzymes on large polymers, were encoded in mobile genetic elements (Richards and Talbot, 2013).

These observations are consistent with the idea of horizontal gene transfer enabling cooperation in a community due to the invasion of mobile elements transmitting cooperative traits. However, the mobile elements also generate a cost to the cells harbouring them and, therefore, can potentially be lost or outcompeted by ‘cheat’ genetic elements (Rankin *et al*., 2011). Recent experimental evidences show nevertheless that horizontal gene transfer helps to maintain the production of public goods despite the potential presence of non‐cooperative organisms and non‐cooperative mobile elements (Dimitriu *et al*., 2015) owing, among other factors, to the increase in genetic relatedness due to the presence of the mobile elements (Mc Ginty *et al*., 2013). In other words, transmissible mobile elements allow for the local enrichment in cooperative interactions, which may, in the long term, lead to the specialization of sub‐populations in cooperative niches specially in the presence of strong structure (Niehus *et al*., 2015).

## The relevance of cooperation for biotechnological applications

The presence of cooperative interactions facilitates the development of complex functions that would be otherwise difficult or impossible (Nowak, 2006).

**Figure 4:**
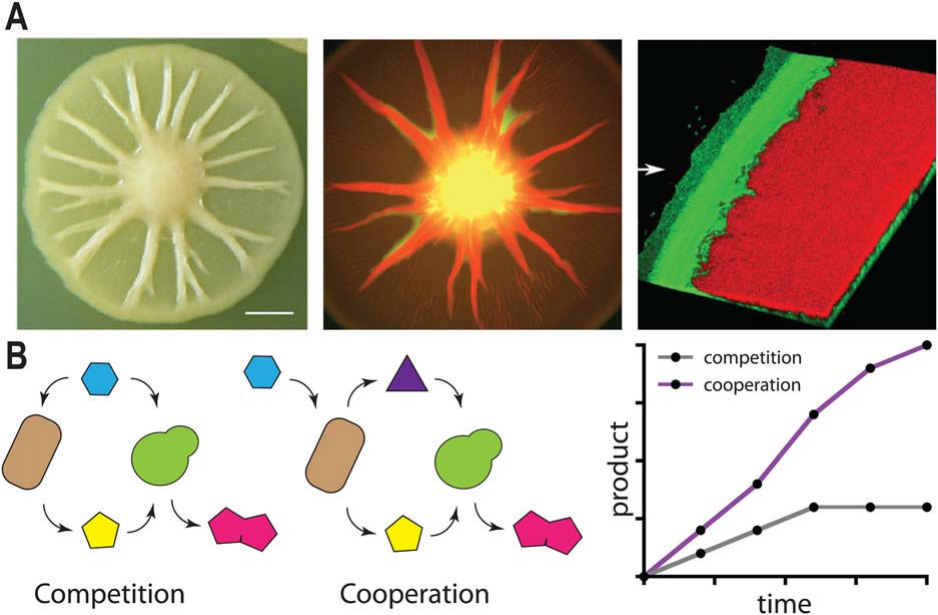
A. Division of labour in microbial populations. Colonies of *Pseudomonas fluorescens* Pf0‐1 are composed by cells with two different morphologies known as mucoid and dry that can evolve from each other due to a single mutation (left picture). Colonies composed by a mixture of the two phenotypes expand faster allowing cells to colonize larger regions in shorter periods of time compared to colonies composed by each of the individual phenotypes. The two morphotypes occupy different regions of the colony as shown when labelled with fluorescent reporters (centre). Dry cells (in red) exhibit a radial distribution growing on top of the mucoid (in green). Confocal microscopy reveals that the edge of the colony (right picture) displays a distinct spatial organization in which mucoid cells form a thin strip at the very edge. The differentiation and spatial segregation allows the distribution of labour in the population: Mucoid cells produce a lubricant polymer at the edge, whereas dry cells sit behind and push both of them along. The cooperation of these two phenotypes results in a fast growing colony. Pictures have been reproduced from Kim *et al*. (2016). B. Engineered populations can improve bioprocesses. Two strains are combined to carry out the synthesis of a product of interest (red pentagons) that cannot be produced using each of the strains individually. The process involves that one strain produces an intermediate (the yellow pentagon) that is used by the other to synthesize the final product. If the two strains compete for the same resources (e.g. carbon source shown by the blue hexagon; left panel) the population with the lower fitness under those conditions will eventually collapse. However, when the two populations are engineered so that one grows at the expenses of the other (e.g. through cross‐feed or syntrophy shown by the purple triangle), the two populations cooperate (centre panel) and the synthesis of the product of interest takes place for a longer period of time resulting in higher yields (right panel). Panels inspired by Zhou *et al*. (2015).

Cooperative microorganisms can exhibit distribution of labour: a large collection of distinct phenotypic behaviours, organized in subpopulations, can coordinate to fulfil some complex tasks in a collective way (Fig. 4A). Shared diffusible molecules allow cells to communicate and spatially distribute the labour. Examples of complex tasks range from the controlled growth of biofilms depending on environmental conditions (Liu *et al*., 2015; Kim *et al*., 2016) to the distributed computation of Boolean functions (Regot *et al*., 2011).

This type of interaction is commonly observed in biodegradative processes carried out by interspecies biofilms. For instance, the presence of a algae in a microbial consortium with more than nine bacterial species enhances the degradation of the pesticide diclofop methyl (Wolfaardt *et al*., 1994). Another interesting example is the syntrophic interaction between the non‐cellulolytic species *Treponema bryantii*, and the cellulolytic species *Ruminococcus flavefaciens*, to enhance the rate of cellulose degradation. The slowly growing cultures of *R. flavefaciens* benefits from *T. bryantii* removing the cellulolytic product, which results in higher population density and degradation rates (James *et al*., 1995).

Distribution of labour is, however, not restricted to spatially structured populations or populations composed by more than one species, but can also apply to other biological processes like the biochemical pathways for the degradation of aromatics in populations composed of one strain (Nikel *et al*., 2014). These pathways are sometimes organized into two distinct gene operons: one encoding for the activities required to funnel the aromatic substrate into a more affordable aromatic carbon source and a second required to transform this aromatic compound into central metabolites. For instance, the TOL pathway of *Pseudomonas putida* responsible for toluene and xylene degradation contains an ‘upper’ part that converts toluene into benzoate, and a ‘lower’ segment responsible for the degradation of benzoate (Franklin *et al*., 1981). In principle, it would be expected that all cells express both operons when a clonal population of *P. putida* is cultured in the presence of toluene but, surprisingly, many of the cells display a near bimodal distribution expressing either one operon or the other (Nikel *et al*., 2014). The mechanistic explanation of this behaviour is unknown although a plausible explanation of the phenotypic distribution may arise from the intricate transcriptional control of the operons (Silva‐Rocha and de Lorenzo, 2012). Distribution of labour also appears in the anaerobic metabolism of aromatic compounds in *Rhodopseudomonas palustris*. Monocultures of this species organize in three different subpopulations when using *p*‐coumarate or benzoate as the carbon source. Each of these subpopulations is responsible for the utilization of either the aromatic compound, CO_2_ and H_2_ or, when growing on benzoate, N_2_ and formate, forming a syntrophic consortia *de facto* composed of a single species (Karpinets *et al*., 2009). However, whether this particular type of cooperative cross‐feeding interaction is advantageous to prevent waste of resources or accumulation of toxic intermediates is an open question.

Distribution of labour can also be engineered together with cooperative traits in ‘synthetic’ communities (Fig. 4B). This is the case of co‐culturing engineered strains of the bacterium *E. coli* and the yeast *S. cerevisiae* that are artificially mutualistic. Each of these strains is modified to express one module of the biosynthetic pathway of an antitumoral compound of interest (the acetylated diol paclitaxel precursor). The cooperation between these species allows production of taxanes with higher yields than using *E. coli* alone. The mixed culture combines the capabilities of *E. coli* for producing the intermediate taxadiene with the superior properties of *S. cerevisiae* to catalyse the oxygenation reactions required to render the final compound (Zhou *et al*., 2015). Synthetic consortia can be used in bioprocesses even in the absence of mutualism as explained in the previous sections (e.g. if eco‐evo feedbacks take place). This is the case of an artificial community designed to produce isobutanol from cellulosic biomass composed by the fungus *Trichoderma reesei* and an engineered strain of *E. coli*. In this consortium *T. reesei* acts as a cooperator secreting cellulases required to degrade lignocellulosic polymers and the resulting saccharides are used to feed the *E. coli* strain that delivers the final product (Minty *et al*., 2013). Synthetic communities can also improve biodegradation processes compared with monocultures. Degradation of crude oil is a good example in which microbial communities can exhibit cooperative interactions in Nature including metabolic cross‐talk and shared goods that may contribute to the formation of interspecies biofilms (McGenity *et al*., 2012). Moreover, these interactions can be harnessed to produce artificial communities with enhanced degradation capabilities suitable for oil removal (Gallego *et al*., 2007). Another example is the desulphurization of dibenzothiophene (DBT) to form sulphur‐free 2‐hydroxybiphenyl. In a recent work, DBT desulphurization was carried out using either an engineered *P. putida* strain expressing all the *dszABCD* genes required in the process, or a mixed culture of the same strain expressing only some of the genes. In this experiment, desulfuration of DBT was higher when combining multiple cells ‘specialising’ in one step of the biochemical pathway compared to the case of having all reactions taking place in the same organism (Martínez *et al*., 2016).

Cooperative interactions in microbial communities can also lead to higher resistance to environmental and ecological stress. Empirical observations using artificial communities of yeast show that this resistance takes place over a wide range of conditions (Gore *et al*., 2009). In addition, experiments carried out with engineered populations of *B. subtilis* lacking the ability to form biofilms show that they nevertheless tend to form clusters that, although can have reduced growth due to limited mobility, allow the cells to endure harsh environmental conditions (Ratzke and Gore, 2016). In this case, cooperative individuals tend to aggregate leading to the ‘privatization’ of public goods and to the exclusion of cheating individuals (Pande *et al*., 2016). On the other hand, the loss of cooperation makes cellular communities more fragile (Sanchez and Gore, 2013) and more vulnerable to compositional shifts arising, for example, from antibiotic treatments (Liu *et al*., 2015). The fact that these behaviours are observed in experiments with different manipulated species suggests that these mechanisms are general and could be commonplace in Nature.

The presence of mechanisms that facilitate cooperation can also lead to complex co‐evolutionary dynamics with the consequent emergence of novel social interactions. The most significant example in this respect is the mechanism of quorum sensing that is involved in controlling the investment in ‘public goods’ (Allen *et al*., 2016). Although the original role of quorum sensing is unknown, its ability to facilitate the (beneficial) presence of cooperative interactions may have led to the selection of complex functionalities, e.g., coordinating the expression of genes involved in multiple cooperative strategies, often co‐evolving with them (Popat *et al*., 2015). This example suggests the possibility of using the presence of cooperative interactions to direct the evolution of the communities towards other properties of interest.

## Conclusion

The key point of evolutionary game theory is that the fitness of individuals depends not only on the environment but also on other members in the population. This theory provides a framework to understand the dynamics of many bioprocesses involving complex microbial populations (natural and synthetic) in which the fitness of an individual cell is in fact affected by the environment and by the presence of other cells. A particular case of this scenario concerns the presence of cooperative interactions based on public goods and metabolic interactions that have been the main focus of this review. We have also discussed some of the factors shaping these interactions such as cellular and thermodynamic constraints, as well as factors stabilising them such as structured environments, feedbacks arising from the ecology of the population, cellular regulatory mechanisms implementing certain behavioural strategies and the role of mobile genetic elements. These properties endow cooperative microbial populations with the possibility to resist cheaters invasions and the capability of performing more sophisticated tasks.

Despite its growing use to study the evolution of cooperation, evolutionary game theory has had so far a very limited impact in field or industrial biotechnological applications in which the environmental conditions are generally not well‐defined and may affect the microbial communities (Bouchez *et al*., 2000; Sayler and Ripp, 2000; Cases and de Lorenzo, 2005). In fact, we have presented several examples suggesting that cooperative interactions based on cross‐feeding and public goods are at the core of many processes relevant for industrial biotechnology including food, energy and environmental applications of microorganisms.

Therefore, they are suitable of improvement by incorporating the mechanisms investigated in the large literature of the evolution of cooperation. As we have discussed, populations could be manipulated based on thermodynamic constrains to promote certain metabolic (cooperative) interactions. Similarly, bioprocesses, including bioreactor design, could be engineered to account (and exploit) for eco‐evo feedbacks and spatial organizations.

Understanding how syntrophy and cooperation endow the microbial populations with resistance and resilience against ecological and environmental disturbances like compositional shifts in the environment or antibiotic shocks could be used to engineer robust microbial communities with enhanced performance and predictable dynamics (Briones and Raskin, 2003; Allison and Martiny, 2008; Sözen *et al*., 2014). Overall, we believe that the migration of results and methodologies from the area of evolutionary game theory into the design of microbial consortia would facilitate the engineering of evolutionary resilient communities with a better performance in a wide range of biotechnological applications.

## Conflict of interest

The authors do not a have conflict of interest to declare.

## References

[emi13767-bib-0001] Ackermann, M. , Stecher, B. , Freed, N.E. , Songhet, P. , Hardt, W.‐D. , and Doebeli, M. (2008) Self‐destructive cooperation mediated by phenotypic noise. Nature 454: 987–990. 18719588 10.1038/nature07067

[emi13767-bib-0002] Adami, C. , Schossau, J. , and Hintze, A. (2016) Evolutionary game theory using agent‐based methods. Phys Life Rev 19: 1–26. 27617905 10.1016/j.plrev.2016.08.015

[emi13767-bib-0003] Allen, R.C. , McNally, L. , Popat, R. , and Brown, S.P. (2016) Quorum sensing protects bacterial co‐operation from exploitation by cheats. ISME J 10: 1706–1716. 26744811 10.1038/ismej.2015.232 PMC4918439

[emi13767-bib-0004] Allison, S.D. (2005) Cheaters, diffusion and nutrients constrain decomposition by microbial enzymes in spatially structured environments. Ecol Lett 8: 626–635.

[emi13767-bib-0005] Allison, S.D. , and Martiny, J.B. (2008) Resistance, resilience, and redundancy in microbial communities. Proc Natl Acad Sci USA 105: 11512–11519. 18695234 10.1073/pnas.0801925105 PMC2556421

[emi13767-bib-0006] Arnesen, S. , Havn Eriksen, S. , Olsen, J. , and Jensen, B. (1998) Increased production of α‐amylase from *Thermomyces lanuginosus* by the addition of Tween 80. Enzyme Microb Technol 23: 249–252.

[emi13767-bib-0007] Avery, S.V. (2006) Microbial cell individuality and the underlying sources of heterogeneity. Nat Rev Microbiol 4: 577–587. 16845428 10.1038/nrmicro1460

[emi13767-bib-0008] Axelrod, R.M. (1990) The Evolution of Cooperation. London: Penguin Books.

[emi13767-bib-0009] Bachmann, H. , Fischlechner, M. , Rabbers, I. , Barfa, N. , Branco dos Santos, F. , Molenaar, D. , and Teusink, B. (2013) Availability of public goods shapes the evolution of competing metabolic strategies. Proc Natl Acad Sci USA 110: 14302–14307. 23940318 10.1073/pnas.1308523110 PMC3761572

[emi13767-bib-0010] Basan, M. , Hui, S. , Okano, H. , Zhang, Z. , Shen, Y. , Williamson, J.R. , and Hwa, T. (2015) Overflow metabolism in *Escherichia coli* results from efficient proteome allocation. Nature 528: 99–104. 26632588 10.1038/nature15765 PMC4843128

[emi13767-bib-0011] Berdugo‐Clavijo, C. , Dong, X. , Soh, J. , Sensen, C.W. , and Gieg, L.M. (2012) Methanogenic biodegradation of two‐ringed polycyclic aromatic hydrocarbons. FEMS Microbiol Ecol 81: 124–133. 22324881 10.1111/j.1574-6941.2012.01328.x

[emi13767-bib-0012] Bickel, P.J. , Hammel, E.A. , and O'Connell, J.W. (1975) Sex bias in graduate admissions: data from Berkeley. Science 187: 398–404. 17835295 10.1126/science.187.4175.398

[emi13767-bib-0013] Bouchez, T. , Patureau, D. , Dabert, P. , Juretschko, S. , Doré, J. , Delgenès, P. , *et al* (2000) Ecological study of a bioaugmentation failure. Environ Microbiol 2: 179–190. 11220304 10.1046/j.1462-2920.2000.00091.x

[emi13767-bib-0014] Briones, A. , and Raskin, L. (2003) Diversity and dynamics of microbial communities in engineered environments and their implications for process stability. Curr Opin Biotechnol 14: 270–276. 12849779 10.1016/s0958-1669(03)00065-x

[emi13767-bib-0015] Burns, R.G. (2010) How do microbial extracellular enzymes locate and degrade natural and synthetic polymers in soil In Molecular Environmental Soil Science at the Interfaces in the Earth's Critical Zone. Xu J., and Huang P.M. (eds). Berlin, Heidelberg: Springer Berlin Heidelberg, pp. 294–297.

[emi13767-bib-0016] Callaghan, A.V. , Morris, B.E.L. , Pereira, I.A.C. , McInerney, M.J. , Austin, R.N. , Groves, J.T. , *et al* (2012) The genome sequence of *Desulfatibacillum alkenivorans* AK‐01: a blueprint for anaerobic alkane oxidation. Environ Microbiol 14: 101–113. 21651686 10.1111/j.1462-2920.2011.02516.x

[emi13767-bib-0017] Carlquist, M. , Fernandes, R.L. , Helmark, S. , Heins, A.‐L. , Lundin, L. , Sørensen, S.J. , *et al* (2012) Physiological heterogeneities in microbial populations and implications for physical stress tolerance. Microb Cell Fact 11: 94. 22799461 10.1186/1475-2859-11-94 PMC3443036

[emi13767-bib-0018] Cases, I. , and de Lorenzo, V. (2005) Genetically modified organisms for the environment: stories of success and failure and what we have learned from them. Int Microbiol 8: 213–222. 16200500

[emi13767-bib-0019] Cavaliere, M. , and Poyatos, J.F. (2013) Plasticity facilitates sustainable growth in the commons. J R Soc Interface 10: 20121006. 23365195 10.1098/rsif.2012.1006 PMC3627111

[emi13767-bib-0020] Chen, S. , Su, L. , Billig, S. , Zimmermann, W. , Chen, J. , and Wu, J. (2010) Biochemical characterization of the cutinases from Thermobifida fusca. J Mol Catal B: Enzym 63: 121–127.

[emi13767-bib-0021] Chuang, J.S. , Rivoire, O. , and Leibler, S. (2009) Simpson's paradox in a synthetic microbial system. Science 323: 272–275. 19131632 10.1126/science.1166739

[emi13767-bib-0022] Coleman, G. , and Elliott, W.H. (1962) Studies on alpha‐amylase formation by *Bacillus subtilis* . Biochem J 83: 256–263. 13880470 10.1042/bj0830256 PMC1243542

[emi13767-bib-0023] Cooper, M.B. , and Smith, A.G. (2015) Exploring mutualistic interactions between microalgae and bacteria in the omics age. Curr Opin Plant Biol 26: 147–153. 26318329 10.1016/j.pbi.2015.07.003

[emi13767-bib-0024] Cueto‐Rojas, H.F. , van Maris, A.J.A. , Wahl, S.A. , and Heijnen, J.J. (2015) Thermodynamics‐based design of microbial cell factories for anaerobic product formation. Trends Biotechnol 33: 534–546. 26232033 10.1016/j.tibtech.2015.06.010

[emi13767-bib-0025] Datta, M.S. , Korolev, K.S. , Cvijovic, I. , Dudley, C. , and Gore, J. (2013) Range expansion promotes cooperation in an experimental microbial metapopulation. Proc Natl Acad Sci USA 110: 7354–7359. 23569263 10.1073/pnas.1217517110 PMC3645579

[emi13767-bib-0026] Dimarogona, M. , Topakas, E. , and Christakopoulos, P. (2012) Cellulose degradation by oxidative enzymes. Comput Struct Biotechnol J 2: e201209015. 24688656 10.5936/csbj.201209015 PMC3962083

[emi13767-bib-0027] Dimitriu, T. , Misevic, D. , Lindner, A.B. , and Taddei, F. (2015) Mobile genetic elements are involved in bacterial sociality. Mob Genet Elements 5: 7–11. 26435881 10.1080/2159256X.2015.1006110 PMC4588217

[emi13767-bib-0028] Doebeli, M. (2002) A model for the evolutionary dynamics of cross‐feeding polymorphisms in microorganisms. Popul Ecol 44: 59–70.

[emi13767-bib-0029] Doyle, E. , Noone, A. , Kelly, C. , Quigley, T. , and Fogarty, W. (1998) Mechanisms of action of the maltogenic α‐amylase of *Byssochlamys fulva* . Enzyme Microb Technol 22: 612–616.

[emi13767-bib-0030] El‐Fallal, A. , Abou, M. , El‐Sayed, A. , and Omar, N. (2012) Starch and microbial α‐amylases: from concepts to biotechnological applications In Carbohydrates – Comprehensive Studies on Glycobiology and Glycotechnology. Chang C.‐F. (ed.). InTech, Rijeka (Croatia).

[emi13767-bib-0031] Enfors, S.O. , Jahic, M. , Rozkov, A. , Xu, B. , Hecker, M. , Jürgen, B. , *et al* (2001) Physiological responses to mixing in large scale bioreactors. J Biotechnol 85: 175–185. 11165362 10.1016/s0168-1656(00)00365-5

[emi13767-bib-0032] Eriksson, A. , and Lindgren, K. (2005) Cooperation driven by mutations in multi‐person Prisoner's Dilemma. J Theor Biol 232: 399–409. 15572064 10.1016/j.jtbi.2004.08.020

[emi13767-bib-0033] Fiegna, F. , and Velicer, G.J. (2003) Competitive fates of bacterial social parasites: persistence and self‐induced extinction of *Myxococcus xanthus* cheaters. Proc Biol Sci 270: 1527–1534. 12965020 10.1098/rspb.2003.2387 PMC1691394

[emi13767-bib-0034] Fiore, C.L. , Longnecker, K. , Kido Soule, M.C. , and Kujawinski, E.B. (2015) Release of ecologically relevant metabolites by the cyanobacterium *Synechococcus elongatus* CCMP 1631. Environ Microbiol 17: 3949–3963. 25970745 10.1111/1462-2920.12899

[emi13767-bib-0035] Flamholz, A. , Noor, E. , Bar‐Even, A. , Liebermeister, W. , and Milo, R. (2013) Glycolytic strategy as a tradeoff between energy yield and protein cost. Proc Natl Acad Sci USA 110: 10039–10044. 23630264 10.1073/pnas.1215283110 PMC3683749

[emi13767-bib-0036] Franklin, F.C. , Bagdasarian, M. , Bagdasarian, M.M. , and Timmis, K.N. (1981) Molecular and functional analysis of the TOL plasmid pWWO from *Pseudomonas putida* and cloning of genes for the entire regulated aromatic ring *meta* cleavage pathway. Proc Natl Acad Sci USA 78: 7458–7462. 6950388 10.1073/pnas.78.12.7458 PMC349287

[emi13767-bib-0037] Frey, E. (2010) Evolutionary game theory: theoretical concepts and applications to microbial communities. Phys A: Stat Mech Appl 389: 4265–4298.

[emi13767-bib-0038] Gallego, J.L.R. , García‐Martínez, M.J. , Llamas, J.F. , Belloch, C. , Peláez, A.I. , and Sánchez, J. (2007) Biodegradation of oil tank bottom sludge using microbial consortia. Biodegradation 18: 269–281. 16821101 10.1007/s10532-006-9061-y

[emi13767-bib-0039] González‐Cabaleiro, R. , Lema, J.M. , Rodríguez, J. , and Kleerebezem, R. (2013) Linking thermodynamics and kinetics to assess pathway reversibility in anaerobic bioprocesses. Energy Environ Sci 6: 3780–3789.

[emi13767-bib-0040] González‐Cabaleiro, R. , Ofiţeru, I.D. , Lema, J.M. , and Rodríguez, J. (2015) Microbial catabolic activities are naturally selected by metabolic energy harvest rate. ISME J 9: 2630–2641. 26161636 10.1038/ismej.2015.69 PMC4817626

[emi13767-bib-0041] Gore, J. , Youk, H. , and van Oudenaarden, A. (2009) Snowdrift game dynamics and facultative cheating in yeast. Nature 459: 253–256. 19349960 10.1038/nature07921 PMC2888597

[emi13767-bib-0042] Großkopf, T. , Consuegra, J. , Gaffé, J. , Willison, J.C. , Lenski, R.E. , Soyer, O.S. , and Schneider, D. (2016) Metabolic modelling in a dynamic evolutionary framework predicts adaptive diversification of bacteria in a long‐term evolution experiment. BMC Evol Biol 16: 163. 27544664 10.1186/s12862-016-0733-x PMC4992563

[emi13767-bib-0043] Großkopf, T. , and Soyer, O.S. (2016) Microbial diversity arising from thermodynamic constraints. ISME J 10: 2725–2733. 27035705 10.1038/ismej.2016.49 PMC5042319

[emi13767-bib-0044] Gross, R.A. , and Kalra, B. (2002) Biodegradable polymers for the environment. Science 297: 803–807. 12161646 10.1126/science.297.5582.803

[emi13767-bib-0045] Harcombe, W.R. , Riehl, W.J. , Dukovski, I. , Granger, B.R. , Betts, A. , Lang, A.H. , *et al* (2014) Metabolic resource allocation in individual microbes determines ecosystem interactions and spatial dynamics. Cell Rep 7: 1104–1115. 24794435 10.1016/j.celrep.2014.03.070 PMC4097880

[emi13767-bib-0046] Harrington, K.I. , and Sanchez, A. (2014) Eco‐evolutionary dynamics of complex social strategies in microbial communities. Commun Integr Biol 7: e28230. 24778764 10.4161/cib.28230 PMC3995729

[emi13767-bib-0047] Hauert, C. , Wakano, J.Y. , and Doebeli, M. (2008) Ecological public goods games: cooperation and bifurcation. Theor Popul Biol 73: 257–263. 18221761 10.1016/j.tpb.2007.11.007 PMC2276362

[emi13767-bib-0048] van Hoek, M.J.A. , and Merks, R.M.H. (2012) Redox balance is key to explaining full vs. partial switching to low‐yield metabolism. BMC Syst Biol 6: 22. 22443685 10.1186/1752-0509-6-22 PMC3384451

[emi13767-bib-0049] Hoh, C.Y. , and Cord‐Ruwisch, R. (1996) A practical kinetic model that considers endproduct inhibition in anaerobic digestion processes by including the equilibrium constant. Biotechnol Bioeng 51: 597–604. 18629824 10.1002/(SICI)1097-0290(19960905)51:5<597::AID-BIT12>3.0.CO;2-F

[emi13767-bib-0050] James, G.A. , Beaudette, L. , and Costerton, J.W. (1995) Interspecies bacterial interactions in biofilms. J Ind Microbiol 15: 257–262.

[emi13767-bib-0051] Jin, Q. , and Bethke, C.M. (2007) The thermodynamics and kinetics of microbial metabolism. Am J Sci 307: 643–677.

[emi13767-bib-0052] Karpinets, T.V. , Pelletier, D.A. , Pan, C. , Uberbacher, E.C. , Melnichenko, G.V. , Hettich, R.L. , and Samatova, N.F. (2009) Phenotype fingerprinting suggests the involvement of single‐genotype consortia in degradation of aromatic compounds by *Rhodopseudomonas palustris* . PLoS One 4: e4615. 19242537 10.1371/journal.pone.0004615 PMC2643473

[emi13767-bib-0053] Kim, W. , Levy, S.B. , and Foster, K.R. (2016) Rapid radiation in bacteria leads to a division of labour. Nat Commun 7: 10508. 26852925 10.1038/ncomms10508 PMC4748119

[emi13767-bib-0054] Knoll, G. , and Winter, J. (1989) Degradation of phenol via carboxylation to benzoate by a defined, obligate syntrophic consortium of anaerobic bacteria. Appl Microbiol Biotechnol 30: 318–324.

[emi13767-bib-0055] Kümmerli, R. , Jiricny, N. , Clarke, L.S. , West, S.A. , and Griffin, A.S. (2009) Phenotypic plasticity of a cooperative behaviour in bacteria. J Evol Biol 22: 589–598. 19170825 10.1111/j.1420-9101.2008.01666.x

[emi13767-bib-0056] Lennon, J.T. , and Denef, V.J. (2015) Evolutionary ecology of microorganisms: from the tamed to the wild In Manual of Environmental Microbiology, 4th Edition. Pillai S.D., Nakatsu C.H., Miller R.V., and Yates M.V. (eds). American Society of Microbiology, Washington, DC. pp. 4.1.2‐1–4.1.2‐12.

[emi13767-bib-0057] Lilja, E.E. , and Johnson, D.R. (2016) Segregating metabolic processes into different microbial cells accelerates the consumption of inhibitory substrates. ISME J 10: 1568–1578. 26771930 10.1038/ismej.2015.243 PMC4918450

[emi13767-bib-0058] Lindemann, S.R. , Bernstein, H.C. , Song, H.‐S. , Fredrickson, J.K. , Fields, M.W. , Shou, W. , *et al* (2016) Engineering microbial consortia for controllable outputs. ISME J 10: 2077–2084. 26967105 10.1038/ismej.2016.26 PMC4989317

[emi13767-bib-0059] Liu, J. , Prindle, A. , Humphries, J. , Gabalda‐Sagarra, M. , Asally, M. , Lee, D.D. , *et al* (2015) Metabolic co‐dependence gives rise to collective oscillations within biofilms. Nature 523: 550–554. 26200335 10.1038/nature14660 PMC4862617

[emi13767-bib-0060] Louca, S. , and Doebeli, M. (2015) Calibration and analysis of genome‐based models for microbial ecology. eLife 4: e08208. 26473972 10.7554/eLife.08208 PMC4608356

[emi13767-bib-0061] Majewski, R.A. , and Domach, M.M. (1990) Simple constrained‐optimization view of acetate overflow in *E. coli* . Biotechnol Bioeng 35: 732–738. 18592570 10.1002/bit.260350711

[emi13767-bib-0062] Martínez, I. , Mohamed, M.E.‐S. , Rozas, D. , García, J.L. , and Díaz, E. (2016) Engineering synthetic bacterial consortia for enhanced desulfurization and revalorization of oil sulfur compounds. Metab Eng 35: 46–54. 26802977 10.1016/j.ymben.2016.01.005

[emi13767-bib-0063] McGenity, T.J. , Folwell, B.D. , McKew, B.A. , and Sanni, G.O. (2012) Marine crude‐oil biodegradation: a central role for interspecies interactions. Aquat Biosyst 8: 10. 22591596 10.1186/2046-9063-8-10 PMC3465203

[emi13767-bib-0064] Mc Ginty, S. , Lehmann, L. , Brown, S.P. , and Rankin, D.J. (2013) The interplay between relatedness and horizontal gene transfer drives the evolution of plasmid‐carried public goods. Proc Biol Sci 280: 20130400. 23760639 10.1098/rspb.2013.0400 PMC3652439

[emi13767-bib-0065] McInerney, M.J. , and Bryant, M.P. (1981) Anaerobic degradation of lactate by syntrophic associations of *Methanosarcina barkeri* and *Desulfovibrio* species and effect of H_2_ on Acetate Degradation. Appl Environ Microbiol 41: 346–354. 16345708 10.1128/aem.41.2.346-354.1981 PMC243697

[emi13767-bib-0066] Minty, J.J. , Singer, M.E. , Scholz, S.A. , Bae, C.‐H. , Ahn, J.‐H. , Foster, C.E. , *et al* (2013) Design and characterization of synthetic fungal‐bacterial consortia for direct production of isobutanol from cellulosic biomass. Proc Natl Acad Sci USA 110: 14592–14597. 23959872 10.1073/pnas.1218447110 PMC3767521

[emi13767-bib-0067] Molenaar, D. , van Berlo, R. , de Ridder, D. , and Teusink, B. (2009) Shifts in growth strategies reflect tradeoffs in cellular economics. Mol Syst Biol 5: 323. 19888218 10.1038/msb.2009.82 PMC2795476

[emi13767-bib-0068] Moreno‐Fenoll, C. , Cavaliere, M. , Martínez‐García, E. , and Poyatos, J.F. (2017) Eco‐evolutionary feedbacks can rescue cooperation in microbial populations. Sci Rep 7: 42561. 28211914 10.1038/srep42561 PMC5304172

[emi13767-bib-0069] Morris, B.E.L. , Henneberger, R. , Huber, H. , and Moissl‐Eichinger, C. (2013) Microbial syntrophy: interaction for the common good. FEMS Microbiol Rev 37: 384–406. 23480449 10.1111/1574-6976.12019

[emi13767-bib-0070] Müller, S. , Harms, H. , and Bley, T. (2010) Origin and analysis of microbial population heterogeneity in bioprocesses. Curr Opin Biotechnol 21: 100–113. 20138500 10.1016/j.copbio.2010.01.002

[emi13767-bib-0071] Nadell, C.D. , Drescher, K. , and Foster, K.R. (2016) Spatial structure, cooperation and competition in biofilms. Nat Rev Microbiol 14: 589–600. 27452230 10.1038/nrmicro.2016.84

[emi13767-bib-0072] Nadell, C.D. , Xavier, J.B. , and Foster, K.R. (2009) The sociobiology of biofilms. FEMS Microbiol Rev 33: 206–224. 19067751 10.1111/j.1574-6976.2008.00150.x

[emi13767-bib-0073] Niehus, R. , Mitri, S. , Fletcher, A.G. , and Foster, K.R. (2015) Migration and horizontal gene transfer divide microbial genomes into multiple niches. Nat Commun 6: 8924. 26592443 10.1038/ncomms9924 PMC4673824

[emi13767-bib-0074] Nikel, P.I. , Silva‐Rocha, R. , Benedetti, I. , and de Lorenzo, V. (2014) The private life of environmental bacteria: pollutant biodegradation at the single cell level. Environ Microbiol 16: 628–642. 24341371 10.1111/1462-2920.12360

[emi13767-bib-0075] Nogueira, T. , Rankin, D.J. , Touchon, M. , Taddei, F. , Brown, S.P. , and Rocha, E.P.C. (2009) Horizontal gene transfer of the secretome drives the evolution of bacterial cooperation and virulence. Curr Biol 19: 1683–1691. 19800234 10.1016/j.cub.2009.08.056 PMC2773837

[emi13767-bib-0076] Nowak, M.A. (2006) Five rules for the evolution of cooperation. Science 314: 1560–1563. 17158317 10.1126/science.1133755 PMC3279745

[emi13767-bib-0077] Nowak, M.A. , and Sigmund, K. (2004) Evolutionary dynamics of biological games. Science 303: 793–799. 14764867 10.1126/science.1093411

[emi13767-bib-0078] Paczia, N. , Nilgen, A. , Lehmann, T. , Gätgens, J. , Wiechert, W. , and Noack, S. (2012) Extensive exometabolome analysis reveals extended overflow metabolism in various microorganisms. Microb Cell Fact 11: 122. 22963408 10.1186/1475-2859-11-122 PMC3526501

[emi13767-bib-0079] Pande, S. , Kaftan, F. , Lang, S. , Svatoš, A. , Germerodt, S. , and Kost, C. (2016) Privatization of cooperative benefits stabilizes mutualistic cross‐feeding interactions in spatially structured environments. ISME J 10: 1413–1423. 26623546 10.1038/ismej.2015.212 PMC5029186

[emi13767-bib-0080] Pennisi, E. (2009) Origins. On the origin of cooperation. Science 325: 1196–1199. 19729633 10.1126/science.325_1196

[emi13767-bib-0081] Pfeiffer, T. , and Bonhoeffer, S. (2004) Evolution of cross‐feeding in microbial populations. Am Nat 163: E126–135. 15266392 10.1086/383593

[emi13767-bib-0082] Popat, R. , Cornforth, D.M. , McNally, L. , and Brown, S.P. (2015) Collective sensing and collective responses in quorum‐sensing bacteria. J R Soc Interface 12. 10.1098/rsif.2014.0882 PMC4305403 25505130

[emi13767-bib-0083] Rankin, D.J. , Rocha, E.P.C. , and Brown, S.P. (2011) What traits are carried on mobile genetic elements, and why? Heredity 106: 1–10. 20332804 10.1038/hdy.2010.24 PMC3183850

[emi13767-bib-0084] Ratzke, C. , and Gore, J. (2016) Self‐organized patchiness facilitates survival in a cooperatively growing *Bacillus subtilis* population. Nat Microbiol 1: 16022. 27572641 10.1038/nmicrobiol.2016.22

[emi13767-bib-0085] Regot, S. , Macia, J. , Conde, N. , Furukawa, K. , Kjellén, J. , Peeters, T. , *et al* (2011) Distributed biological computation with multicellular engineered networks. Nature 469: 207–211. 21150900 10.1038/nature09679

[emi13767-bib-0086] Richards, T.A. , and Talbot, N.J. (2013) Horizontal gene transfer in osmotrophs: playing with public goods. Nat Rev Microbiol 11: 720–727. 24018383 10.1038/nrmicro3108

[emi13767-bib-0087] Ross‐Gillespie, A. , Gardner, A. , Buckling, A. , West, S.A. , and Griffin, A.S. (2009) Density dependence and cooperation: theory and a test with bacteria. Evolution 63: 2315–2325. 19453724 10.1111/j.1558-5646.2009.00723.x

[emi13767-bib-0088] Sanchez, A. , and Gore, J. (2013) Feedback between population and evolutionary dynamics determines the fate of social microbial populations. PLoS Biol 11: e1001547. 23637571 10.1371/journal.pbio.1001547 PMC3640081

[emi13767-bib-0089] Sayler, G.S. , and Ripp, S. (2000) Field applications of genetically engineered microorganisms for bioremediation processes. Curr Opin Biotechnol 11: 286–289. 10851144 10.1016/s0958-1669(00)00097-5

[emi13767-bib-0090] Schink, B. (1997) Energetics of syntrophic cooperation in methanogenic degradation. Microbiol Mol Biol Rev 61: 262–280. 9184013 10.1128/mmbr.61.2.262-280.1997 PMC232610

[emi13767-bib-0091] Schoener, T.W. (2011) The newest synthesis: understanding the interplay of evolutionary and ecological dynamics. Science 331: 426–429. 21273479 10.1126/science.1193954

[emi13767-bib-0092] Scholten, J.C. , and Conrad, R. (2000) Energetics of syntrophic propionate oxidation in defined batch and chemostat cocultures. Appl Environ Microbiol 66: 2934–2942. 10877789 10.1128/aem.66.7.2934-2942.2000 PMC92094

[emi13767-bib-0093] Seitz, H.J. , Schink, B. , and Conrad, R. (1988) Thermodynamics of hydrogen metabolism in methanogenic cocultures degrading ethanol or lactate. FEMS Microbiol Lett 55: 119–124.

[emi13767-bib-0094] Silva‐Rocha, R. , and de Lorenzo, V. (2012) Stochasticity of TOL plasmid catabolic promoters sets a bimodal expression regime in *Pseudomonas putida* mt‐2 exposed to m‐xylene. Mol Microbiol 86: 199–211. 22845424 10.1111/j.1365-2958.2012.08184.x

[emi13767-bib-0095] Sözen, S. , Çokgör, E.U. , Başaran, S.T. , Aysel, M. , Akarsubaşı, A. , Ergal, I. , *et al* (2014) Effect of high loading on substrate utilization kinetics and microbial community structure in super fast submerged membrane bioreactor. Bioresour Technol 159: 118–127. 24632634 10.1016/j.biortech.2014.02.003

[emi13767-bib-0096] Spencer, C.C. , Bertrand, M. , Travisano, M. , and Doebeli, M. (2007) Adaptive diversification in genes that regulate resource use in *Escherichia coli* . PLoS Genet 3: e15. 17238290 10.1371/journal.pgen.0030015 PMC1779306

[emi13767-bib-0097] Travisano, M. , and Velicer, G.J. (2004) Strategies of microbial cheater control. Trends Microbiol 12: 72–78. 15036323 10.1016/j.tim.2003.12.009

[emi13767-bib-0098] Van Dyken, J.D. , Müller, M.J.I. , Mack, K.M.L. , and Desai, M.M. (2013) Spatial population expansion promotes the evolution of cooperation in an experimental Prisoner's Dilemma. Curr Biol 23: 919–923. 23664975 10.1016/j.cub.2013.04.026 PMC4405629

[emi13767-bib-0099] Velicer, G.J. , and Vos, M. (2009) Sociobiology of the *Myxobacteria* . Annu Rev Microbiol 63: 599–623. 19575567 10.1146/annurev.micro.091208.073158

[emi13767-bib-0100] Vemuri, G.N. , Altman, E. , Sangurdekar, D.P. , Khodursky, A.B. , and Eiteman, M.A. (2006) Overflow metabolism in *Escherichia coli* during steady‐state growth: transcriptional regulation and effect of the redox ratio. Appl Environ Microbiol 72: 3653–3661. 16672514 10.1128/AEM.72.5.3653-3661.2006 PMC1472329

[emi13767-bib-0101] West, S.A. , Diggle, S.P. , Buckling, A. , Gardner, A. , and Griffin, A.S. (2007) The social lives of microbes. Annu Rev Ecol Evol Syst 38: 53–77.

[emi13767-bib-0102] West, S.A. , Griffin, A.S. , Gardner, A. , and Diggle, S.P. (2006) Social evolution theory for microorganisms. Nat Rev Microbiol 4: 597–607. 16845430 10.1038/nrmicro1461

[emi13767-bib-0103] Westerholm, M. , Dolfing, J. , Sherry, A. , Gray, N.D. , Head, I.M. , and Schnürer, A. (2011) Quantification of syntrophic acetate‐oxidizing microbial communities in biogas processes. Environ Microbiol Rep 3: 500–505. 23761313 10.1111/j.1758-2229.2011.00249.x PMC3659410

[emi13767-bib-0104] Widder, S. , Allen, R.J. , Pfeiffer, T. , Curtis, T.P. , Wiuf, C. , Sloan, W.T. , *et al* (2016) Challenges in microbial ecology: building predictive understanding of community function and dynamics. ISME J 10: 2557–2568. 27022995 10.1038/ismej.2016.45 PMC5113837

[emi13767-bib-0105] Wierckx, N. , Prieto, M.A. , Pomposiello, P. , de Lorenzo, V. , O'Connor, K. , and Blank, L.M. (2015) Plastic waste as a novel substrate for industrial biotechnology. Microb Biotechnol 8: 900–903. 26482561 10.1111/1751-7915.12312 PMC4621443

[emi13767-bib-0106] Willsey, G.G. , and Wargo, M.J. (2015) Extracellular lipase and protease production from a model drinking water bacterial community is functionally robust to absence of individual members. PLoS One 10: e0143617. 26599415 10.1371/journal.pone.0143617 PMC4657875

[emi13767-bib-0107] Wolfaardt, G.M. , Lawrence, J.R. , Robarts, R.D. , and Caldwell, D.E. (1994) The role of interactions, sessile growth and nutrient amendments on the degradative efficiency of a microbial consortium. Can J Microbiol 40: 331–340. 8069778 10.1139/m94-055

[emi13767-bib-0108] Yoshida, S. , Hiraga, K. , Takehana, T. , Taniguchi, I. , Yamaji, H. , Maeda, Y. , *et al* (2016) A bacterium that degrades and assimilates poly(ethylene terephthalate). Science 351: 1196–1199. 26965627 10.1126/science.aad6359

[emi13767-bib-0109] Zamocky, M. , Ludwig, R. , Peterbauer, C. , Hallberg, B.M. , Divne, C. , Nicholls, P. , and Haltrich, D. (2006) Cellobiose dehydrogenase – a flavocytochrome from wood‐degrading, phytopathogenic and saprotropic fungi. Curr Protein Pept Sci 7: 255–280. 16787264 10.2174/138920306777452367

[emi13767-bib-0110] Zhou, K. , Qiao, K. , Edgar, S. , and Stephanopoulos, G. (2015) Distributing a metabolic pathway among a microbial consortium enhances production of natural products. Nat Biotechnol 33: 377–383. 25558867 10.1038/nbt.3095 PMC4867547

[emi13767-bib-0111] Zhuang, K. , Vemuri, G.N. , and Mahadevan, R. (2011) Economics of membrane occupancy and respiro‐fermentation. Mol Syst Biol 7: 500. 21694717 10.1038/msb.2011.34 PMC3159977

[emi13767-bib-0112] Zomorrodi, A.R. , and Segrè, D. (2016) Synthetic ecology of microbes: mathematical models and applications. J Mol Biol 428: 837–861. 26522937 10.1016/j.jmb.2015.10.019 PMC4798899

